# Pathogenesis of Type 2 Epithelial to Mesenchymal Transition (EMT) in Renal and Hepatic Fibrosis

**DOI:** 10.3390/jcm5010004

**Published:** 2015-12-30

**Authors:** Anusha H. Tennakoon, Takeshi Izawa, Mitsuru Kuwamura, Jyoji Yamate

**Affiliations:** 1 Laboratory of Veterinary Pathology, Life and Environmental Sciences, Osaka Prefecture University, Rinkuu Ourai Kita 1-58, Izumisano, Osaka 598-8531, Japan; tennakoonah@gmail.com (A.H.T.); izawa@vet.osakafu-u.ac.jp (T.I.); kuwamura@vet.osakafu-u.ac.jp (M.K.); 2 Teaching Hospital Peradeniya, Peradeniya 20400, Sri Lanka

**Keywords:** epithelial to mesenchymal transition, renal fibrosis, hepatic fibrosis, animal models, myofibroblasts, hepatic progenitor cells, bile ductular reaction

## Abstract

Epithelial to mesenchymal transition (EMT), particularly, type 2 EMT, is important in progressive renal and hepatic fibrosis. In this process, incompletely regenerated renal epithelia lose their epithelial characteristics and gain migratory mesenchymal qualities as myofibroblasts. In hepatic fibrosis (importantly, cirrhosis), the process also occurs in injured hepatocytes and hepatic progenitor cells (HPCs), as well as ductular reaction-related bile epithelia. Interestingly, the ductular reaction contributes partly to hepatocarcinogenesis of HPCs, and further, regenerating cholangiocytes after injury may be derived from hepatic stellate cells via mesenchymal to epithelia transition, a reverse phenomenon of type 2 EMT. Possible pathogenesis of type 2 EMT and its differences between renal and hepatic fibrosis are reviewed based on our experimental data.

## 1. Introduction

Epithelial to mesenchymal transition (EMT), where epithelial cells lose their epithelial nature and gain mesenchymal characteristics, has been given much attention in the life science community [1]. The first description of possible EMT was proposed in 1982 [2]. Thus far, the pivotal roles of EMT have been seen in both physiological and pathological conditions; EMT plays roles in tissue modeling or remodeling [3,4]. EMT is basically classified into three types based on different biological settings with different functional consequences [1,5]. Type 1 EMT occurs during normal organogenesis; the generated immature mesenchymal cells form more mature epithelia via the mesenchymal to epithelial transition (MET); although type 1 is a converse phenomenon to EMT in pathological fibrosis, type 1 is important for investigating the other EMT in pathological settings (types 2 and 3), as mentioned below. Type 2 EMT is associated with tissue repair responses such as fibrosis to underlying injuries in parenchymal organs. Type 2 EMT gives rise to myofibroblasts from epithelia to heal injured tissues; if the injury is mild and acute, the healing process is regarded as reparative fibrosis; on the contrary, in ongoing chronic inflammation, abnormal formation of myofibroblasts cause progressive fibrosis, thereafter leading to organ parenchymal destruction by excessive extra-cellular matrix (ECM) deposition. Type 3 EMT is related to malignancy, where neoplastic cells can migrate into surrounding tissues and invade at metastasis sites; this EMT occurs in carcinomas derived from epithelial cells, in which neoplastic epithelial cells are transformed into cells with mesenchymal nature. In all three types of EMT, there are various molecular events, including transcription factor activation, specific cell surface protein expression, reorganization of cytoskeletal proteins, production of ECM degradation enzymes and changes of specific microRNAs [1]. However, distinct differences governing the three distinct EMT types are not fully understood.

Over the last two decades, using human pathological tissues and experimental animal models, knowledge of EMT has accumulated. In this review, to shed some light on this subject, we focus on type 2 EMT in renal and hepatic fibrosis based on our experimental data using animal models. Basically, using cisplatin (CDDP)-induced rat renal fibrosis model, we have demonstrated further significance for EMT in renal fibrosis; in this animal model, possible participations of transforming growth factor-β1 (TGF-β1), platelet derived growth factor (PDGF)-BB, prostaglandins, osteopontin (OPN), neutrophil gelatinase-associated lipocalin (NGAL) and bone morphogenic protein-6 (BMP-6) are discussed. For hepatic fibrosis, we used thioacetamide (TAA) to induce liver fibrosis/cirrhosis model, focusing on the relation between hepatic progenitor cells (HPCs) and EMT. In addition to TAA-induced hepatic cirrhosis, the roles of EMT were investigated in α-naphthylisothiocyanate (ANIT)-induced rat biliary fibrosis occurring in the Glisson’s sheath.

## 2. Pathogenesis of Type 2 Epithelial to Mesenchymal Transition (EMT) in Renal Fibrosis

### 2.1. Renal Fibrosis and Disease Models

Chronic kidney disease, characterized by extensive interstitial fibrosis, has become a major worldwide healthcare burden [6]. Irrespective of the etiology, renal fibrosis is the final common pathway of progressive kidney diseases [7]. Myofibroblasts, the cells morphologically and functionally intermediate between fibroblasts and smooth muscle cells, are the main source of excessive ECM deposition in renal fibrosis. Derivation of renal myofibroblasts may be heterogeneous; renal epithelia/endothelia, interstitial fibroblastic cells or mesenchymal pericytes have been proposed and, of these, the EMT process from injured renal epithelia is regarded as the most important pathway leading to formation of interstitial myofibroblasts in diseased kidneys at advanced stages [8,9,10]. Nephrogenesis consists of mutual induction of two cell populations derived from intermediate mesoderm: epithelial cells of the ureteric bud that grow out from the metanephric duct, and mesenchymal cells of the metanephric mesenchyme [11,12]. The Glomeruli and renal tubules, except collecting ducts are developmentally derived from the metanephric mesenchyme through MET (type 1 EMT) [13]. Injury to adult organs recapitulates embryonic programming in remodeling [14]. Thus, injured epithelia in chronic renal disease are thought to undergo regression to the metanephric mesenchymal phenotype and acquire myofibroblastic cell characteristics through EMT, showing reverse embryogenesis [15,16,17]. However, the significance of EMT in renal fibrosis is not fully understood [18], particularly on the formation of myofibroblastic cells via type 2 EMT.

Cultured tubular epithelial cell lines (such as NRK-52E) are frequently used to demonstrate type 2 EMT [19,20,21,22]. On the contrary, the occurrence of type 2 EMT in experimentally-induced renal failure models is inconsistent and, depending on the experimental conditions, some models are discovered to be EMT-prone and others EMT-resistant [23]. In unilateral ureteric obstruction (UUO) model, pressure-induced damage leads to progressive interstitial fibrosis via type 2 EMT phenomenon [24,25,26,27]. Renal fibrosis induced in rats by cisplatin (CDDP), a widely used anti-cancer drug with renal toxicity, is considered to be the best model of post-tubular injury fibrosis [28,29]. CDDP-induced renal lesions are characterized histopathologically by necrosis or desquamation of proximal renal epithelia and subsequent dilatation of the affected renal tubules [29,30]. The affected epithelia have the capacity of regeneration; however, completely damaged basal lamina causes incomplete regeneration leading to interstitial fibrosis via type 2 EMT, as abnormal regenerating renal epithelia show positive reaction to α-smooth muscle actin (α-SMA), an immunophenotypical maker of myofibroblasts as mentioned below [30,31]. Compared to UUO models with frequent α-SMA-positive renal epithelial cells, type 2 EMT in CDDP-induced rat renal fibrosis is less frequent; the pressure-induced damage tends to induce more frequently type 2 EMT in kidney lesions.

### 2.2. Expression of Type 2 EMT Markers

During the process of type 2 EMT, cell–cell interactions of epithelia are lost with repressed tight junction proteins such as claudin and occludin, and then epithelia obtain the elongated migratory mesenchymal morphology of myofibroblasts [32]. Although the process is very complicated, the expression of cell surface protein such as cadherins and integrins is used to monitor type 2 EMT [33]; there is a downregulation of epithelial *E*-cadherin and upregulation of mesenchymal *N*-cadherin, so-called cadherin switch. Additionally, the expression of cytoskeletal markers, particularly α-SMA, vimentin, desmin, fibroblast specific protein-1 (FSP-1) and β-catenin, has been evaluated to characterize renal tubular epithelia undergoing type 2 EMT [5,34,35]. Concomitant expression of both epithelial and mesenchymal markers indicates the presence of cells in the intermediate stages via type 2 EMT. α-SMA, a component of actin cytoskeleton, is a well-accepted marker for completed myofibroblasts to identify the EMT process in injured renal epithelia [36,37]. Reorganization of α-SMA-expressing cells undergoing EMT in both renal tubules and surrounding interstitial fibrotic areas may imply cell elongation and directional motility through disrupted basement membrane [32,38]. α-SMA acts as stress fibers in myofibroblasts augmenting their contractile ability and migration, which is critical for tissue remodeling [33]. α-SMA-positive myofibroblasts can produce ECMs such as collagens and fibronectin, culminating in renal interstitial fibrosis and then scar formation (contracted kidneys) [30,39].

In addition to α-SMA, different cytoskeletal proteins expressed in myofibroblasts are regarded as useful markers for myofibroblast differentiation [30]. Vimentin has frequently been seen not only in type 2 EMT but also in type 3 EMT in carcinoma cells [40,41] and desmin expression is seen in human and mouse podocytes after glomerular damage (type 2 EMT) [42]. Using CDDP- and UUO-induced rat fibrotic kidneys, we have demonstrated expression of α-SMA and vimentin in both renal epithelia and interstitial cells, supporting the usefulness of these two markers to recognize type 2 EMT-undergoing cells [30]. Although interstitial myofibroblastic cells in spontaneous canine fibrotic kidneys expressed α-SMA, epithelia in the fibrotic lesions did not show α-SMA. However, vimentin expression was more strongly and frequently seen in both renal epithelia and interstitial cells in the fibrotic lesions of canine kidneys [30]. Desmin expression was seen only in interstitial myofibroblasts of CDDP-treated rat kidneys and epithelia of canine fibrotic kidneys, indicating that desmin is not always useful as a marker of EMT in both rat and canine fibrotic kidneys [30]. These results indicate interspecies heterogeneity of cytoskeletal immunoexpression, which should be considered when interpreting type 2 EMT of renal epithelia in fibrotic kidneys [30].

### 2.3. Growth Factors Associated with Type 2 EMT

Type 2 EMT is triggered by a variety of soluble factors. The most powerful factor is TGF-β [43]. Out of three family members (TGF-β1, -β2, and -β3), TGF-β1 plays a critical role in type 2 EMT [44,45]. TGF-β1 exerts its effect through either Smad or non-Smad pathway, and when added to cultured renal epithelial cell lines, the epithelia change from cuboidal to fusiform in shape and acquire myofibroblastic mesenchymal nature with repressed epithelial markers [32,46,47]. Similarly, we confirmed that a rat mesenchymal immature mesenchymal cell line MT-9 (with pericyte-like nature) and a porcine proximal renal epithelial cell line, LLC-PK1, showed a dose-dependent increment of α-SMA after TGF-β1 treatment [30]. In the same experiment, we illustrated that the addition of PDGF-BB, another fibrogenic factor [48], to the above cell lines increased the α-SMA-positive cell number, although the α-SMA expression degree was much less than that under TGF-β1 treatment. Simultaneous addition of TGF-β1 and PDGF-BB to LLC-PK1 showed a greater increment of α-SMA-positive cell number than did the sole addition of TGF-β1, indicating the additive effect of PDGF-BB in TGF-β1-induced type 2 EMT. PDGF-BB may up-regulate TGF-β1 synthesis with common signaling pathways, denoting a possible underlining mechanism for this augmentation [49]. Importance of TGF-β1 has been reported in type 3 EMT in carcinomas, and that of PDGF-BB factor has been demonstrated in the induction of type 3 EMT in carcinoma and type 1 EMT in coronary smooth muscle differentiation [50,51,52]. PDGF-BB involvement in pericytes to myofibroblast conversion of type 2 is demonstrated in UUO and ischemic reperfusion injury in mouse kidneys [53]. In addition to TGF-β1, collectively, studies on PDGF-BB on EMT would be beneficial to understand the molecular mechanisms behind renal type 2 EMT.

### 2.4. Roles of Prostaglandins

Prostaglandins, the lipid autacoids derived from arachidonic acid, play an important role in the pathophysiology of kidneys [54,55]. The factors influence the formation and loss of intercellular contacts in epithelial tissues [56], implying possible participation in type 2 EMT. Significant increment of prostaglandin E2 (PGE2) is observed in tubulointerstitial fibrosis induced by UUO [57,58,59]. PGE2 is the major prostanoid produced in the kidney and thought to influence cell proliferation and differentiation through its receptors, EP2 or EP4 [55,60,61]. PGE2 is synthesized by the conversion of arachidonic acid via cyclooxygenases (COX), COX-1 or COX-2, and terminal prostaglandin E synthases (PGES) [62,63]. Experiments using COX-1 and COX-2 deficient mice have indicated that COX-2 is essential for normal renal development, whereas no alterations were seen in renal structures of COX-1 deficient mice [64,65]. Cyclin D1, a factor of the G1 phase of the cell cycle, is important in cell cycle regulation [66]; its nuclear expression is seen in the G1 phase of the cell cycle and thereafter, the shift into cytoplasmic expression of cyclin D1 means transition to the S phase [67,68]. In CDDP-induced rat renal fibrosis, we have shown participation of endogenous PGE2 in abnormal regeneration of renal tubular epithelia exclusively through EP4 where cyclin D1 expression was restricted within the nucleus of regenerating renal epithelial cells indicating G1 arrest. Interestingly, in the CDDP-induced rat renal fibrosis model, there was decreased COX-2 expression and increased COX-1 expression [69]. In nephrogenesis, in contrast, COX-2 expression is increased, accompanied by cyclin D1 expression both in the nucleus and cytoplasm of developing renal tubules [70]. These results indicate that, in addition to the importance of PGE2 in both normal and abnormal renal tubular development via EP4, COX-1 may play in more crucial roles in abnormal regeneration of renal tubules in CDDP-induced rat renal fibrosis. Although it is thought that the regeneration process of injured renal tubules is analogous to embryogenesis, these results indicate that there are differences between renal tubular development and regeneration of renal tubules; abnormal regeneration of injured renal tubules should be responsible for type 2 EMT. That is, incomplete regeneration of renal epithelial cells after renal damage can lead to progressive interstitial fibrosis, probably via EMT [31]. The addition of EP4 receptor agonist to cultured rat renal epithelial cell line, NRK-52E, reduces the expression of TGF-β1-induced α-SMA expression, indicating the inhibition of EMT [69]. Similar results were demonstrated using EP4^−/−^ mice *in vivo* after UUO [59]. On the other hand, in a prostate carcinoma model, type 3 EMT was inhibited by EP4 antagonism [61]. These results indicate that the same molecule, EP4 (a receptor of PGE2), has different roles in type 2 and type 3 EMT.

### 2.5. Neutrophil Gelatinase-Associated Lipocalin (NGAL), Osteopontin (OPN) and Bone Morphogenic Protein-6 (BMP-6)

NGAL, a lipocalin superfamily protein, was first identified in activated neutrophils [71]. Later, its expression was identified in epithelia in inflammatory lesions and in malignancy [72]. NGAL expression is upregulated after damaged renal epithelia; therefore, its expression is regarded as a promising tubular biomarker in the diagnostics of acute kidney diseases, both in clinical and experimental settings [73,74,75]. OPN is an acidic glycoprotein synthesized in bone and various epithelial tissues; its expression is limited in the loop of Henle and distal tubules of normal rat kidneys, whereas the upregulated expression is seen in all renal tubule segments after renal injury [76,77]. OPN has multifunctional roles in bone morphogenesis, macrophage infiltration and tumorigenesis [77,78]. In CDDP-induced rat renal fibrosis, NGAL expression was seen in completely regenerating proximal renal tubules with regularly arranged epithelial cells, correlating well with proliferating activity. Interestingly, OPN expression was seen in dilated or atrophied abnormal renal tubules surrounded by flattened or irregularly-arranged epithelia, around which interstitial fibrosis was taking place; the increased expression of OPN significantly correlated with α-SMA-positive myofibroblast appearance, expression of TGF-β1 mRNA and CD68-positive macrophages [79,80]. Treatment of NRK-52E with TGF-β1 decreased NGAL expression, whereas OPN expression was increased; furthermore, *E*-cadherin was decreased but α-SMA expression was increased. It is considered that NGAL is involved in favorable regeneration of renal tubules after injury, whereas OPN expressing in incomplete regeneration of renal epithelia participates in renal progressive fibrosis via type 2 EMT. In fact, the addition of OPN to NRK-52E induces EMT [81]. However, there is a report describing that NGAL may participate in type 3 EMT in carcinoma with metastasis [82,83], an opposite phenomenon to type 2 EMT in renal fibrosis.

**Scheme 1:**
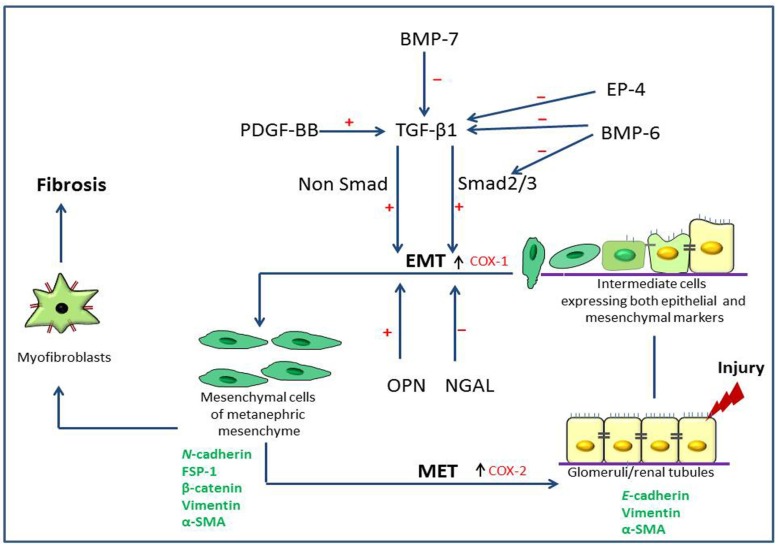
Possible epithelial to mesenchymal transition mechanisms of renal fibrosis. Mesenchymal cells of metanephric mesenchyme give rise to renal epithelial cells during embryogenesis through the mesenchymal to epithelial transition (MET), and these cells express epithelial markers such as *E*-cadherin and mesenchymal cell markers such as vimentin and α-smooth muscle actin (α-SMA). After injury, renal epithelial cells undergo phenotypical changes through the epithelial to mesenchymal transition (EMT, type 2), in which they acquire intermediate phenotypes expressing both epithelial and mesenchymal markers; they further transform into mesenchymal cells (expressing mesenchymal markers such as *N*-cadherin, fibroblast specific protein-1 (FSP-1), β-catenin, vimentin and α-SMA). EMT is considered the reverse embryogenesis of MET. Finally, these mesenchymal cells become myofibroblasts which are responsible for progressive renal fibrosis. During the MET process, there is an increment of cyclooxygenase (COX)-2, whereas during EMT, COX-1 increases. Transforming growth factor-β1 (TGF-β1) generated via non-Smad and Smad pathways stimulates the EMT in renal fibrosis. Platelet derived growth factor-BB (PDGF-BB) has an additive effect on the TGF-β1-induced EMT. Prostaglandin receptor 4 (EP4), bone morphogenic protein-6 (BMP-6) and neutrophil gelatinase-associated lipocalin (NGAL) have inhibitory effects on type 2 EMT. Bone morphogenic protein-7 (BMP-7) counteracts TGF-β1-induced EMT [94]. (+, stimulation; −, inhibition; ↑, increment).

BMP-6 is a member of TGF-β superfamily [84]. Generally, it is known that the TGF-β superfamily plays pivotal roles in renal fibrosis by antagonizing TGF-β-induced pro-fibrogenic signaling [85]. TGF-β exerts its functions mainly through its down stream signaling molecules, Smads 2 and 3 [86]. Particularly, the anti-fibrotic effect of BMP-7 is well documented and has been given attention as a possible therapeutic target [87,88,89]. BMP-7 has an inhibitory action, particularly on Smad 3 [90], and exerts its effects by reducing ECM deposition by inactivating ECM-producing myofibroblasts and EMT, and by enhancing ECM degradation [91]. Similarly, BMP-6 deficiency aggravate interstitial damage and fibrosis in UUO mouse model independent of BMP-7 [92]. In CDDP-induced rat renal fibrosis, BMP-6 expression was seen in abnormal renal epithelial cells and also in peri-tubular myofibroblasts in CDDP-induced rat renal fibrosis [80]. The addition of TGF-β1 to NRK-52E increased expression of BMP-6; on the contrary, BMP-6 treatment decreased TGF-β1 expression of NRK-52E cells. BMP-6 has an anti-fibrotic effect by the inhibition of TGF-β1 activity by suppressing TGF-β1-induced JNK activation and Smad signaling [93]. Additionally, it might have a direct role on TGF-β1 functions. Besides BMP-7, BMP-6 would be another possible therapeutic tool [93]. Taken together, the possible participation of type 2 EMT in kidney fibrosis is shown in Scheme 1.

Apart from TGF-β1, studying the roles of PDGF-BB on the induction of EMT would be beneficial to understand the underling molecular mechanisms of renal type 2 EMT. Further studies on the dual roles of EP4 and NGAL between type 2 (favorable effects) and type 3 EMT (negative effects) would lead to better understanding the underscoring mechanisms governing different types of EMT. BMP-6 and BMP-7 might emerge as a possible new therapeutic tool to improve progressive renal fibrosis.

## 3. Pathogenesis of Type 2 Epithelial to Mesenchymal Transition (EMT) in Hepatic Fibrosis

### 3.1. Liver Fibrosis and Disease Models

Irrespective of the cause, dysregulated wound healing response in the liver with excessive ECM deposition results in progressive cirrhosis, which is characterized by the formation of regenerative and degenerative nodules (pseudolobules) separated by fibrous septa [95,96,97]. Liver has a massive regenerating capability after injury with restoring its lost mass and adjusting the size to that of the organism [98]. Despite this fascinating ability of regeneration, liver cirrhosis has become a leading cause of death worldwide. Hepatitis B and C viral infection, auto-immune diseases, alcohol abuse and metabolic diseases such as non-alcoholic fatty liver disease and non-alcoholic steatohepatitis (NASH) are causes of hepatic cirrhosis [99,100,101]. Additionally, hepatic cirrhosis has become a main cause of hepatocellular carcinoma. To study hepatic cirrhosis, various animal models have been utilized [102]. We have used toxin-induced rat models: thioacetamide (TAA)-induced and α-naphthylisothiocyanate (ANIT)-induced hepatic fibrosis; the former develops centrilobular injury/necrosis and subsequent fibrosis, the latter induced peri-biliary fibrosis in the Glisson’ sheath [103,104]. The TAA-induced rat hepatic cirrhosis, induced by repeated injections, bears a close resemblance to human micro-nodular cirrhosis [96,105,106]. ANIT injection to rats damages bile duct epithelial cells, thereby producing intrahepatic cholestasis, bile duct hyperplasia (bile duct reaction) and peri-biliary fibrosis [107,108,109].

### 3.2. Possible Liver EMT in Thioacetamide (TAA)-Induced Rat Cirrhosis

After transient tissue injury, reparative fibrosis occurs, whereas persistent and repeated damage induces progressive fibrosis, leading to cirrhosis. The fibrotic lesions is characterized by deposition of ECM produced by myofibroblasts [99]. Hepatic myofibroblasts are heterogeneous in origin and nature. Quiescent hepatic stellate cells (HSCs) are considered to be the major source of hepatic myofibroblasts. Additionally, bone marrow stem cells, pre-existing fibroblasts and mesenchymal cells via type 2 EMT of hepatocytes or bile duct epithelia have been proposed as other derivation of hepatic myofibroblasts [110,111]. However, EMT as a source of hepatic myofibroblasts is highly controversial [112,113,114]. TGF-β1-treated mouse hepatocytes showed downregulation of *E*-cadherin and upregulation of mesenchymal marker (such as vimentin) and type I collagen synthesis, indicating possible EMT *in vitro* [115]. *In vivo* evidence for hepatocyte EMT was illustrated by Zeisberg and colleagues using a double transgenic mouse model where hepatocytes that undergo EMT contribute to the FSP1-positive fibroblasts in carbon tetrachloride-induced liver fibrosis [116]. In addition to hepatocytes, biliary epithelia could give rise to hepatic myofibroblasts through type 2 EMT. Evidence for biliary epithelia EMT was shown in a bile duct ligation (BDL)-induced mouse hepatic fibrosis [117], and possible contribution of cholangiocytes to fibrosis via type 2 EMT was demonstrated *in vitro* [118]. The co-localization of CK19 (a marker of bile ductular cells) and mesenchymal markers such as FSP-1 and vimentin has been demonstrated in samples of human biliary atresia and in cultures of hepatic progenitor cells (HPCs) [119,120]. HPCs are cells capable of differentiating into hepatocytes and bile duct epithelia. Proliferation and expansion of HPCs located in the canals of Herring, so-called “ductular reaction”, always occurs in the vicinity of myofibroblasts in fibrotic lesions, indicating possible involvement of type 2 EMT of HPCs [121,122,123]. In studies using TAA-induced rat liver cirrhosis, we observed HPC-related bile duct reactions depended on progressive fibrosis. Expression of glial fibrillary acidic protein (GFAP) (a marker for activated HSCs/hepatic myofibroblasts) and cytokeratin 19 (CK19) (a marker for bile duct cells and HPCs) was observed simultaneously in reacting bile duct cells and HPCs [103]. Additionally, GFAP-expressing myofibroblasts in rat cirrhotic livers were present, raising the possibility of type 2 EMT either via bile duct cells or HPCs. In contrast to observation by Xia and coworkers in BDL-mouse model [117], however, no co-expression of α-SMA (the well accepted hepatic myofibroblast marker) and CK19 was observed in reacting bile duct cells and HPCs in TAA-induced rat cirrhosis; furthermore, there was no cadherin switch (from *E*-cadherin to *N*-cadherin) in these ductular cells with progressive cirrhosis. There was also no immunohistochemical evidence for type 2 EMT. Recapitulation of embryogenesis in fibrosis is a key indication for type 2 EMT. In the kidney, tubular epithelium is of mesodermal origin derived from intermediate mesoderm via MET (type 1 EMT). Therefore, renal tubular epithelia could retain their mesenchymal imprints and return easily to a mesenchymal state via type 2 EMT during renal fibrosis after injury. On the other hand, in the liver, all the epithelia are derived from the foregut endoderm [1,124]; therefore it is unlikely to revert to a mesenchymal phenotype via type 2 EMT during hepatic fibrogenesis.

### 3.3. Importance of Ductal Reaction and Possible Hepatocarcinogenesis, Instead of Type 2 EMT, in TAA-Induced Rat Cirrhosis

As mentioned above, type 2 EMT of bile ducts or HPCs is very unlikely. Interestingly, it is thought that the ductular reaction in hepatic cirrhosis is a recapitulation of hepatic ontogenesis [125]. The ductal plate, generated from the embryonic precursor cells, remodels to form intrahepatic bile ducts and periportal hepatocytes during embryogenesis [126]. The remnants of the ductal plate give rise to the canals of Herring in adult liver, of which cells produce bi-potential HPCs in liver lesions [127]. Particularly, in cirrhotic liver, this cell compartment expands, forming ductular reaction; although such event is regarded as an effort to restore the normal hepatic architecture, novel hepatocytes and bile duct cells are developed as the result. In TAA-induced rat hepatic cirrhosis, the analysis of gene profiles related to ductular reaction by laser microdissection demonstrated higher expression of TGF-β1 and PDGF-β mRNA in the HPC locality [103]. These factors. upregulated in ductal epithelia. have shown to activate transition of surrounding HSCs to hepatic myofibroblasts, leading to hepatic fibrosis [123]. The intimate association of HPCs and myofibroblasts in cirrhotic livers is not related to type 2 EMT phenomenon. Additionally, we have showed that, in TAA-induced rat cirrhosis, there was greater mRNA expression of both Wnt2 and Wnt4 which act via canonical and non-canonical Wnt signaling pathways, respectively; furthermore, increased expression of Glypican-3, which belongs to the family of heparan sulfate proteoglycans and is reported to promote the growth of hepatocellular carcinoma [128,129], was seen [103]. The Wnt/β-catenin pathway is also involved not only in liver embryogenesis but also in activation of tumorigenic HPCs [130]. Because HPCs are thought to be a possible precursor of hepatocellular carcinomas, the increased expression of these genes in ductular reaction the vicinity of HPCs may imply possible development of hepatocellular carcinomas at the advanced stages of hepatic cirrhosis probably via maturation arrest of HPCs [131]. In hepatic cirrhosis, the bile duct reaction (ductular reaction), which are derived from HPCs, should be considered to have different roles in tumorigenesis, although the bile duct reaction may be related partly to type 2 EMT leading to progressive fibrosis [132,133,134].

It is worth mentioning that the immunohistochemical method of concomitant expression of both epithelial and mesenchymal markers has limitations in investigating type 2 EMT in hepatic cirrhosis. Mesenchymal cells are dynamic in their phenotype, and the transforming epithelial cells might not yet have fully activated the expression of mesenchymal genes [3,135]. Therefore, there are somewhat difficulties in observing a transient event of cells both expressing epithelial and mesenchymal markers. Lineage tracing experiments could be beneficial; however, such experiments using different animal hepatic fibrosis models did not demonstrate clear evidence of type 2 EMT of cholangiocytes [136].

### 3.4. Regenerating Cholangiocytes Do Not Induce Type 2 EMT in α-Naphthylisothiocyanate (ANIT)-Induced Peri-Biliary Fibrosis

We considered that peri-biliary fibrosis would be more useful for investigating type 2 EMT. As mentioned above, injection of ANIT in rats can induce peri-biliary fibrosis after cholangiocyte injury. In this ANIT-induced peri-biliary fibrosis, regenerating cholangiocytes after injury showed positive reaction to vimentin and nestin (a type VI intermediate filament protein expressed mainly by neuronal stem cells), in addition to CK19 expression [104]. However, dual reactions reacting to CK19 and α-SMA were not seen in ANIT-induced peri-biliary fibrosis. These findings denied that regenerating cholangiocytes are associated with type 2 EMT. Interestingly, HSCs expressed nestin. Therefore, co-expression of CK19 and nestin in regenerating cholangiocytes in the peri-biliary fibrosis may indicate that HSCs are a possible progenitor of repopulating cholangiocytes after injury. The possible differentiation of HSCs to hepatocytes has been reported [137,138]. Nestin could have a role in the migration of cholangiocyte progenitors, perhaps from nestin-positive HSCs in the periportal area or vimentin/nestin-positive connective tissue cells in the Glisson’s sheath [104]. Reactivity for Ki67, a marker of cellular proliferation, in HSCs co-expressing nestin, vimentin and CK19 indicates that subpopulation of HSCs might differentiate into cholangiocytes by the migration [104,138]. Hence, in ANIT-induced peri-biliary fibrosis, nestin-expressing mesenchymal HSCs may be possible progenitor of repopulating cholangiocytes, indicating the MET; this EMT may be a reverse phenomenon of type 2 EMT [104].

### 3.5. Type 2 EMT and Hepatic Cirrhosis

**Scheme 2:**
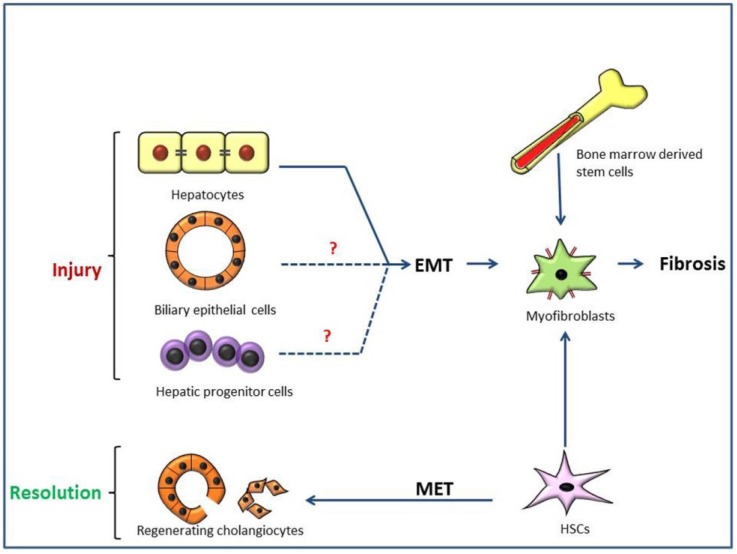
Possible epithelial to mesenchymal transition (EMT) mechanisms of liver fibrosis. Hepatic stellate cells (HSCs), bone marrow-derived stem cells and mesenchymal cells via type 2 EMT from hepatocytes, biliary epithelial cells or hepatic progenitor cells are depicted as the possible sources of myofibroblasts in progressive liver fibrosis (cirrhosis) [110,111,116,117,121,122,123]. The experiments focusing on EMT of biliary epithelia and hepatic progenitor cells show no evidence supporting the process. However, in the resolution phase of biliary fibrosis, HSCs could undergo mesenchymal to epithelial transition (MET), giving rise to regenerating cholangiocytes. (?, inconclusive evidences).

In TAA-induced cirrhosis and ANIT-induced peri-biliary fibrosis, collectively, there was no clear evidence of type 2 EMT via biliary epithelia or HPCs. However, we showed that ductal reaction in TAA-induced cirrhosis may be related to possible hepatocarcinogenesis of HPCs, and that regenerating cholangiocytes in ANIT-induced peri-biliary fibrosis may be derived from HSCs via MET, a reverse phenomenon of type 2 EMT (Scheme 2).

## 4. Conclusions

EMT is a critical process that occurs both in normal development as well as in pathological settings. In this review, we focused on type 2 EMT, which is related to renal and hepatic fibrosis. There were differences in significance and occurrence of type 2 EMT-related fibrosis between the kidney and liver. It is worth exploring the underlying molecular mechanisms to find any differences. Furthermore, comparing different types of EMT (types 1, 2 and 3) would lead to better understanding of EMT, a unique phenomenon of the body. Progressive fibrosis in the kidney and liver is an intractable disease. The clarification of the pathogenesis of type 2 EMT would provide very useful information for possible therapies and expanding knowledge on stem cell biology would open up novel dimensions in understanding the process.
